# Addressing noncommunicable diseases among urban refugees in the Middle East and North Africa - a scoping review

**DOI:** 10.1186/s13031-020-0255-4

**Published:** 2020-02-18

**Authors:** Zahirah Z. McNatt

**Affiliations:** 1 Department of Community Health and Social Medicine, University of Global Health Equity, Kigali Heights, Plot 772, KG 7 Ave., 5th floor, P.O. Box 6955, Kigali, Rwanda; 2 Mailman School of Public Health, Department of Population and Family Health, Columbia University, 60 Haven Avenue B3, New York, 10032 USA

**Keywords:** Non-communicable disease, Chronic disease, Health systems, Refugees, Humanitarian response, Syria, Middle East and North Africa, Urban, Semi-urban

## Abstract

**Background:**

More than 5.5 million Syrian refugees have fled violence and settled in mostly urban environments in neighboring countries. The Middle East and North Africa (MENA) region accounts for 6% of the global population but 25% of the population are ‘of concern’ to the UN Refugee Agency. In addition to large amounts of forced migration, the region is also undergoing an epidemiologic transition towards a heavier burden of noncommunicable diseases (NCDs), which in 2018 accounted for 74% of all deaths in the region. To address NCD needs globally, a myriad of policies and interventions have been implemented in low-income stable country settings. However, little is known about which policies and interventions are currently being implemented or are best suited for refugee hosting countries across the Middle East and North Africa.

**Methods:**

A scoping review of peer-reviewed literature was conducted to identify policies and interventions implemented in the Middle East and North Africa to address the needs of urban refugees with noncommunicable diseases.

**Results:**

This scoping review identified 11 studies from Jordan, Lebanon, Iran, West Bank, Gaza and Syria. These studies addressed three foci of extant work, (1) innovative financing for expensive treatments, (2) improvements to access and quality of treatment and, (3) efforts to prevent new diagnoses and secondary complications. All interventions targeted refugee populations including Palestinians, Sudanese, Syrians, Afghans and Iraqis.

**Discussion:**

The scoping review highlighted five key findings. First, very few studies focused on the prevention of noncommunicable diseases among displaced populations. Second, several interventions made use of health information technologies, including electronic medical records and mHealth applications for patients. Third, the vast majority of publications were solely focused on tackling NCDs through primary care efforts. Fourth, the literature was very sparse in regard to national policy development, and instead favored interventions by NGOs and UN agencies. Last, the perspectives of refugees were notably absent.

**Conclusion:**

Opportunities exist to prioritize prevention efforts, scale up eHealth interventions, expand access to secondary and tertiary services, address the scarcity of research on national policy, and incorporate the perspectives of affected persons in the broader discourse.

## Background

More than 5.5 million Syrian refugees have fled violence and conflict, and settled in mostly urban environments in neighboring countries. The Middle East and North Africa (MENA) accounts for 6% of the global population but comprises 25% of the ‘population of concern’ to the UN Refugee Agency (including refugees, internally displaced persons, asylum-seekers and stateless persons). The vast majority of refugees in the MENA region live outside of camp settings in urban and semi-urban environments in Jordan, Lebanon and Turkey [1–3]. In addition to large numbers of displaced persons in urban settings, the region is also undergoing an epidemiologic transition towards more noncommunicable diseases (NCDs). In 2018, NCDs accounted for 74% of deaths in the Middle East and North Africa. In Lebanon, NCDs accounted for 84% of deaths, 76% in Jordan and 78% in Saudi Arabia. Prior to the civil war in Syria, NCDs accounted for 77% of all deaths and as of 2019, a significant portion of the displaced Syrian population resides in Jordan, Lebanon and Turkey [4, 5]. The dual dynamic of a large number of refugees in urban settings and a high burden of NCDs has placed significant pressure on surrounding low-and middle-income host countries, their health systems and humanitarian actors. These pressures limit the ability of health and humanitarian actors to provide care that is accessible, equitable and high in quality.

As a result, Syrians, as well as other refugee groups, face significant challenges as they receive new NCD diagnoses, manage their illnesses, attempt to access host country health systems and cope with conflict and displacement [6, 7]. The most commonly explored NCDs among the Syrian refugee community in Lebanon and Jordan include hypertension, chronic respiratory diseases, diabetes, arthritis and cardiovascular disease. Hypertension prevalence among Syrian refugees in Jordan and Lebanon was estimated at 9.7 and 7.4%, respectively; diabetes prevalence was 5.3 and 3.3%, respectively; and chronic respiratory disease prevalence was 3.1 and 3.8%, respectively [8, 9]. However, these figures are likely underestimates due to self-report bias and difficulties identifying representative samples. Other refugee populations, including Iraqis, Afghans and Palestinians, also have high NCD burdens. Screening activities across the region estimated that 18% of Palestinian refugees had hypertension, while self-reported hypertension among Iraqi refugees ranged between 3 and 30% [6].

To address NCD needs, a myriad of policies and interventions have been implemented in low-income, stable country settings [10]. In 2010 the World Health Organization (WHO) released The Package of Essential Noncommunicable (PEN) Disease Interventions for Primary Health Care in Low-Resource Settings [11, 12]. The PEN included interventions for heart attack and stroke care as well as asthma and chronic obstructive pulmonary disease (COPD). It provided health education and counseling tools and recommendations for how to develop early diagnosis systems. The PEN prioritized the integration of NCD care into primary health care centers and highlighted key medicines and technologies that should be made available in low-resource settings [11, 12]. In 2017 WHO also released an updated version of “Tackling NCDs: Best buys and other recommended interventions for the prevention and control of NCDs [13]. However, both the PEN and the Best Buys did not address the NCD needs or care priorities in complex humanitarian emergencies, including those that result in the forced migration of large populations into urban settings.

In humanitarian settings, it is important to have better information related not only to the scale and nature of NCD needs but also to useful policies and interventions that support effective, equitable practice. In an attempt to identify NCD interventions implemented in humanitarian settings, Ruby et al. [10] conducted a systematic review of the effectiveness of NCD interventions in humanitarian crises. The authors identified eight studies, the majority of which did not explore interventions in refugee crises. Of the studies that did address refugee needs, all were focused on one intervention for Palestine refugees. However, the Palestinian population is served by the United Nations Relief and Works Agency for Palestine Refugees in the Near East (UNRWA) and thus has a different healthcare access experience than Syrians or any other refugee group in the MENA region. None of the articles included in the review focused on Syrian, Afghan, Iraqi or any other refugee group present in the region.

As a result, little is known about what policies and interventions are currently being implemented for the diverse group of refugees residing in urban settings in low-and middle-income host countries across the Middle East and North Africa [10, 14]. Accordingly, the purpose of this study was to utilize a scoping review methodology to identify policies and interventions aimed at addressing the needs of urban refugees diagnosed with noncommunicable diseases in MENA region.

## Methods

This review was guided by Arksey and O’Malley’s [15] methodological framework for conducting a scoping review. The scoping review methodology was selected to broadly map policies and interventions. This methodology was preferred over that of a systematic review because the literature on this subject is in its infancy and a systematic review would limit the focus to specific study designs and require an assessment of the quality of each study. Scoping reviews allow for the inclusion of all study designs, including basic descriptions of policies and interventions. These descriptions frequently lack an evaluation, but may nonetheless provide valuable information and be significant to understanding the landscape. Arksey and O’Malley’s framework outlined five steps, (1) identifying the research question, (2) identifying relevant studies, (3) selecting studies, (4) charting the data and (5) collating, summarizing and reporting the results [15].

### Research question

The focus of this review was to identify and explore the policies and interventions, implemented by humanitarian actors and host countries, that aim to address NCDs among urban-based refugees in the Middle East and North Africa. The World Health Organization definition for the term “policy” was utilized, stated as the “decisions, plans, and actions that are undertaken to achieve specific health care goals within a society.” [16] The term “urban” in the refugee context refers to cities and towns and excludes refugee camps [17]. Studies were included if urban settings were the focus per a statement in the methods section.

### Identifying relevant studies

To identify relevant studies, four databases--PubMed, EMBASE, Medline and PsychInfo--were searched in November, 2018 for articles published in English between 2000 and 2018. The search strategy combined Medical Subject Headings (MeSH terms; see Table 1) and the following keywords or phrases, (1) non-communicable diseases OR chronic diseases OR diabetes OR hypertension OR cardiovascular disease OR chronic respiratory disease OR cancer AND, (2) refugees, AND the (3) Middle East OR North Africa OR Lebanon OR Turkey OR Jordan OR Iran (see Additional file 1). The search terms were purposefully broad in order to capture all relevant policies and interventions. The most prevalent NCDs and the nations hosting the largest numbers of refugees were added to the search order to capture articles that did not directly use the terms “NCD” or “Middle East and North Africa.” The bibliographies of all studies identified in this previous step were reviewed for related articles. The author also hand-searched reference lists of related articles.

**Table 1:** Search terms

Refugees	Noncommunicable disease	Middle East
	Chronic disease	North Africa
	Diabetes	Lebanon
	Hypertension	Jordan
	Chronic respiratory disease	Turkey
	Cardiovascular disease	Iran
	Cancer	

### Study selection

**Fig. 1:**
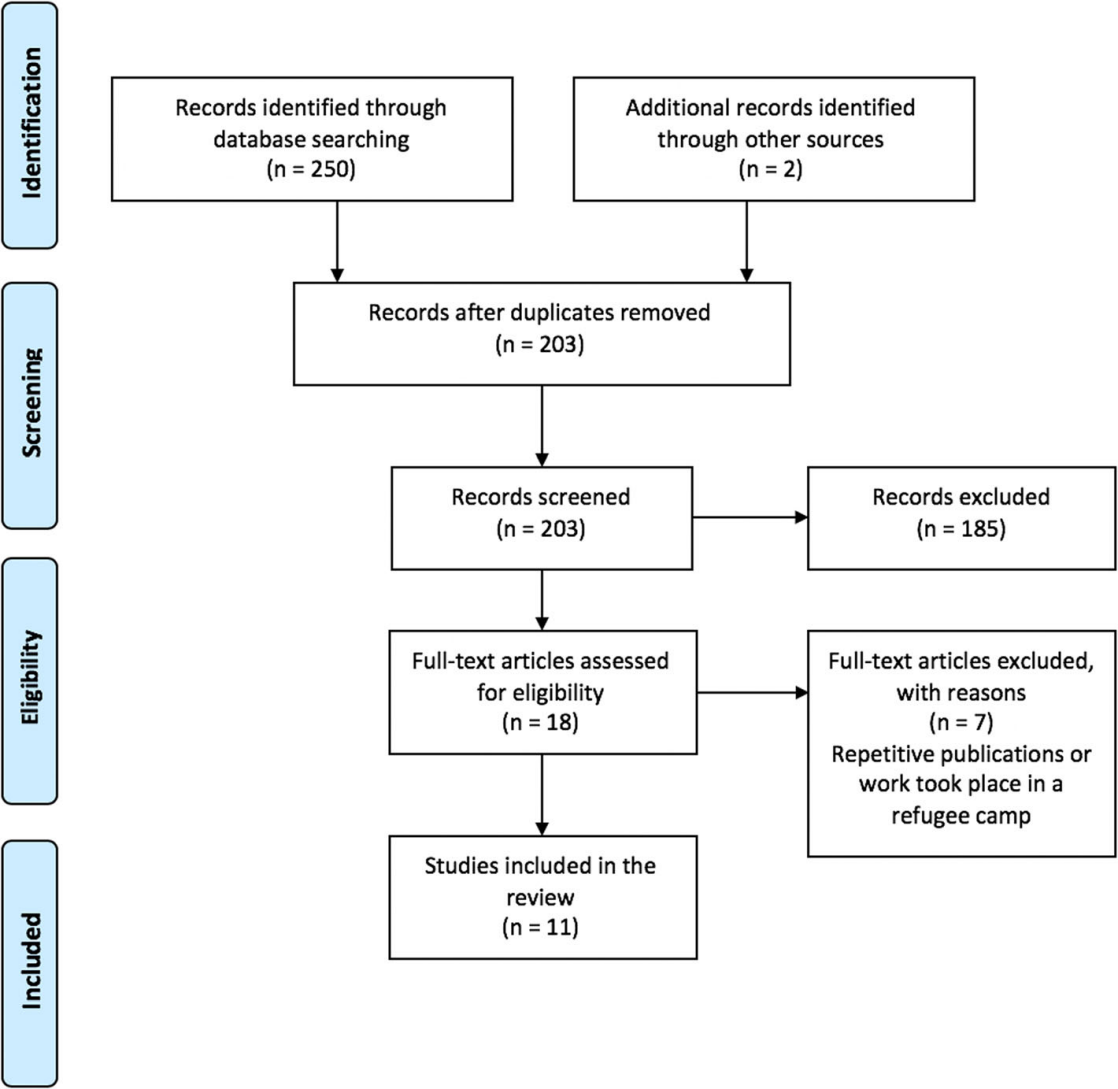
PRISMA diagram: the search and selection process

The search identified a total of 252 articles, including 49 duplicates, which were removed. The remaining articles (203) were eligible for inclusion if they described a policy or intervention that was aimed at addressing NCDs among urban-based refugees in the MENA region. The author reviewed all titles and abstracts and excluded articles based on several criteria (see Table 2). First, when numerous articles relied on the same dataset, only the article(s) which most thoroughly described the approach was included. Second, articles were excluded if they discussed NCDs in high-income countries or focused on prevalence and risk factors, rather than actions taken to improve access and service delivery. Third, opinion pieces, commentaries, news articles and dissertations were excluded. One study was unavailable in the public domain and was replaced by a publication on an earlier version of the same intervention, resulting in 18 relevant articles eligible for full text review. After the full text review, 7 articles were excluded because they focused on refugees residing in refugee camps, rather than urban settings, were repetitive publications or addressed national needs but did not incorporate refugees. Eleven studies were ultimately included (see Fig. 1).

**Table 2:** Inclusion and exclusion criteria

Study characteristics	Inclusion criteria	Exclusion criteria
Population	Refugees in urban settings.	Populations other than urban refugees.
Intervention or policy	Any NCD focused intervention implemented for refugees by a humanitarian actor or a host country health system. Any NCD relevant policy that applied to refugee populations.	Publications/reports that did not describe an intervention or policy. Repetitive publications that presented the same intervention/policy.
Setting	Urban/non-camp environments in the Middle East and North Africa.	High income countries; outside the Middle East and North Africa. Refugee camp settings.
Study design	All study designs.	Opinion pieces, commentaries, dissertations, news articles.
Publications (peer-reviewed)	English language only.	Languages other than English.

### Charting data and collation

Data were extracted from the selected publications and placed in an excel spreadsheet noting the following key variables: author, date of publication, country, aim of the intervention/policy, target population, intervention/policy characteristics, NCDs addressed, how the intervention/policy was measured, outcomes and the implementing organization.

### Analyzing, collating, summarizing and reporting findings

The selected studies were read and reread by the author, who used inductive analysis to identify common themes or categories. The author’s search for themes was also guided by two WHO conceptual frameworks. The first framework identified four core components of NCD care -- prevention, treatment, rehabilitation and palliative care [18]. The second, the Health Systems Framework [19] presented six building blocks of a health system and noted financing as key to the functioning of a health system. The resulting three themes of prevention, treatment and innovative financing (see below) were identified in the scoping review and were well aligned with two significant WHO frameworks that aid in understanding NCDs and health systems.

### Geographic scope

For the purposes of this review, the term MENA was used in alignment with UNHCR’s regional definition, which includes: Algeria, Bahrain, Egypt, Iraq, Israel, Jordan, Kuwait, Lebanon, Libya, Mauritania, Morocco, Oman, Qatar, Saudi Arabia, State of Palestine, Syria, Tunisia, United Arab Emirates, Western Sahara Territory and Yemen. Turkey and Iran were also included in this analysis as a result of their significant involvement in the refugee response.

## Results

The results are based on 11 peer-reviewed articles from Jordan (*N* = 5), Lebanon (*N* = 3), Iran (*N* = 1) and the broader region (*N* = 2). These 11 papers explored the implementation and/or the evaluation of policies and interventions aimed at tackling NCDs among refugees residing in urban/non-camp settings in MENA region (see Table 3). One publication was a mixed-methods study, one was a longitudinal cohort study, five were descriptive studies and one was a non-control, descriptive intervention study. Three were detailed descriptions of interventions with little or no analysis or measurement of effectiveness. The NCDs examined in these publications included Type II diabetes, hypertension, cardiovascular disease, cancer and end-stage renal disease (ESRD). In addition to the policies and interventions implemented in Jordan, Lebanon and Iran, the United Nations Relief and Works Agency for Palestine Refugees in the Near East (UNRWA) implemented regional activities across four or five country settings (West Bank, Gaza, Jordan, Lebanon and Syria). Only one of the 11 papers described a national policy, while the remaining presented interventions that were implemented by NGOs or UN agencies.

**Table 3:** Summary of selected studies

Author and year	Country	Aim of intervention/policy	Target population	Intervention/policy characteristics	NCD(s) addressed	How intervention/policy was measured	Outcomes of intervention / policy* (excerpts from abstracts)	Implementing organization
Ballout et al. (2018) [22]	Jordan, Lebanon, West Bank, Gaza	Improve quality of all service with attention to increase in NCD burden	Palestinian refugees	PHC reform, e-health (EMR) system, appointment system and Family Health Teams.	NCDs	Daily consultations, physician satisfaction, waiting time for patient registration, antibiotic prescription rate	Physician's daily consultations were reduced from 104 to 85. 89% of doctors expressed satisfaction concerning timesaving and efficiency of e-Health. Average wait time in registration queue decreased from 25 minutes to 12 minutes. Average registration time reduced from 6 minutes to 1.5 minutes. Average antiobiotic prescription rate decreased from 27% to 24.5% and average number of medical consultations per day decreased 104 to 85.	United Nations Relief and Works Agency
Collins et al. (2017) [28]	Jordan	Identify cardiovascular disease risk among patient population	Syrian refugees and Jordanians	Cardiovascular disease risk assessment and management tool for physicians in outpatient NCD clinics.	Cardiovascular disease	Mixed methods: demographics, laboratory testing, risk factor measurements, prescribing behavior	23.3% of patients had a documented WHO/ISH risk score of which 65% were correct. 60.4% of patients were eligible for lipid-lowering treatment and 48.3% of these patients were prescribed it. Analysis of interviews with sixteen MSF staff identified nine explanatory themes.	Medecins Sans Frontieres
Doocy et al. (2017) [25]	Lebanon	Improve quality and continuity of care, health literacy, mobility of medical records and health outcomes.	Syrian refugees, Lebanese	Treatment guidelines: Introduction of standards guidelines, training for clinicians, counseling of patients and mHealth: Patient-controlled health record, EMR & decision tool for clinicians.	Hypertension, Type II diabetes	Clinical measurements, patient-provider interaction, medication prescription and use	Recording of BP readings and blood sugar measurements significantly decreased following the implementation of treatment guidelines. Recording of BP readings also decreased after the mHealth phase as compared with baseline. Recording of BMI reporting increased at the end of the mHealth phase from baseline and the guidelines phase. Only differences in BMI were statistically significant. Data extracted from the mHealth app showed that a higher proportion of providers offered lifestyle counseling compared with the counseling reported in patients' paper records. There were statistically significant increases in all four measures of patient-provider interaction across study phases.	International Organization for Migration; International Medical Corps in 10 health centers
Ghods et al. (2005)* [27]	Iran	Make dialysis and kidney transplantation available to refugees	Afghan refugees, Iranians	Integration of refugees into national dialysis and renal transplant program.	End-stage renal disease	Descriptive analysis: # on dialysis, # undergone transplantation, nationality of donors and recipients	Outcomes were not measured	Transplantation Unit, Hashemi Nejad Kidney Hospital
Khader et al. (2012) [23]	Jordan	Inform and improve the quality of health services	Palestinian refugees	Cohort monitoring of hypertension patients using e-health	Hypertension	Descriptive analysis of routine program data: number of patients, patient demographics, clinical measurements	Outcome analysis indicated deficiencies in several components of clinical performance related to blood pressure measurements and fasting blood glucose tests. Between 8% and 15% of patients with HT had serious complications such as cardiovascular disease and stroke.	United Nations Relief and Works Agency
Khader et al. (2012) [24]	Jordan	Inform and improve the quality of health services	Palestinian refugees	Cohort monitoring of diabetes patients using e-health	Diabetes	Descriptive analysis of routine program data: number of patients, patient demographics, clinical measurements	Outcome analysis indicated deficiencies in several components of care: measurement of blood pressure, assessments for foot care, blood tests for glucose, cholesterol and renal function. 10-20% of patients with DM in the different cohorts had serious late complications such as blindness and stroke.	United Nations Relief and Works Agency
Rowther et al. (2015) [29]	Jordan	Prevent diabetes among high-risk patients attending clinic for other illnesses	Syrian, Palestinian, Iraqi refugees and Jordanians	Computer assisted diabetes risk assessment & self-administered motivational interviewing module with one-month telephone follow-up by a nurse.	Type II diabetes	Intervention was not measured	Outcomes were not measured	Institute of Famiy Health (IFH); Noor Al Hussein Foundation; UC Irvine
Saab et al. (2018) [21]	Lebanon	Provide care free of charge for patients and families	Displaced children: Syrian, Palestinian. Non-displaced: Families traveling from Iraq and Syria. Lebanese children.	Funding scheme to support displaced children with cancer.	Cancer	Descriptive analysis: demographics, clinical information, actual & projected budgets, outcomes (including relapse and death)	575 non-Lebanese children suspected to have cancer were evaluated. Of those, 311 received direct medical support, with 107 receiving full-treatment coverage and 204 receiving limited-workup/specialty services; the remaining 264 patients received medical consultations.	American University of Beirut Medical Center; Children's Cancer Center of Lebanon Foundation; St. Jude Children's Research Hospital; American Lebanese Syrian Associated Charities
Abu Kishk et al. (2015). [30] 2018 not available	Jordan, Lebanon, West Bank, Gaza	To encourage behavior change among health center patients	Palestinian refugees	Education, cooking and exercise sessions for patients with type I and II diabetes from 8 health centers	Type I and Type II diabetes	Non-control interventional descriptive study: Analysis of weight, BMI, waist circumference, blood sugar, blood pressure, cholesterol and patient knowledge and behavior	Significant reductions in body measures (i.e., BMI) and biomarkers (i.e., blood pressure)	United Nations Relief and Works Agency
Sethi et al. (2017) [26]	Lebanon	Implement community based primary care for refugees	Syrian refugees	Provide community-based primary care and health promotion through Refugee Outreach Volunteers (also known as CHWs)	Hypertension, diabetes	Summary of initial program efforts: # of visits to monitor blood pressure, capillary glucose and medication adherence; # of refugees referred to PHC and # of home visits for education	Outcomes were not measured	Medical Teams International
Spiegel et al. (2014) [20]	Jordan, Syria	Provide funding for refugees with serious medical conditions	Registered refugees in Jordan and Syria. including Iraqi, Syrian, Sudanese.	Funding scheme to support refugees with serious medical problems. Committee of physicians that makes clinical funding decisions. Exceptional care committees (ECC).	Cancer	Descriptive analysis: demographics, types of cancers, approvals and funding, reasons for denial	Outcomes were not measured	United Nations Relief and Works Agency

### Intervention/policy characteristics

These interventions and policies addressed three main categories, (1) innovative financing for expensive treatments (two papers), (2) improvements to access and quality of treatment (six papers), and (3) efforts to prevent new cases as well as secondary complications (three papers). All interventions targeted refugee populations, such as Palestinians, Sudanese, Syrians, Afghans and Iraqis. Five interventions also focused on host-communities including Jordanians, Iranians and Lebanese. A diversity of actors led these NCD interventions, including NGOs (Médecins Sans Frontières, Institute for Family Health, International Medical Corps, Medical Teams International), UN agencies (UNRWA, the UN Refugee Agency, International Organization for Migration) and health facilities (i.e., American University of Beirut Medical Center, Hashemi Nejad Kidney Hospital).

#### Theme 1: financing cancer treatment for adults and children

In two studies, the purpose of the interventions was to provide funding for displaced persons with cancer in Jordan, Syria and Lebanon. Spiegel et al. (2014) described one funding mechanism solely managed by UNHCR, the Exceptional Care Committee (ECC), which required individuals to apply for funds to cover cancer treatments in Jordan and Syria [20]. Saab et al. [21] described a novel funding collaborative between two medical centers in Beirut, Lebanon, and Memphis, Tennessee, respectively [21]. Both publications stated that resource-poor settings and expensive treatments were barriers to cancer care. The authors also noted that financial burdens were exacerbated by a lack of insurance coverage for refugees and other displaced persons. Specifically, Saab et al. [21] stated that while care for Lebanese patients was expensive, most Lebanese patients had partial insurance coverage, thus reducing the financial burden on the medical center partners. Both interventions were able to finance care for cancer treatment; however, the funds available were often insufficient [20, 21]. Between 2011 and 2017, 311 non-Lebanese children received treatment as a result of the medical center collaboration. However, only 107 of them received full treatment. The remaining only had partial treatment covered by the medical center collaboration in Lebanon. In Jordan, UNHCR’s Exceptional Care Committee (ECC) received 511 applications for funding for cancer treatment (between 2010 and 2012) but could only fund 246, which is less than 50% of the requests [20, 21]. These funding mechanisms supported the secondary and tertiary needs of a small segment of patients.

#### Theme 2: improving access to high quality NCD care

Six of the 11 selected articles focused on improving access to NCD care and/or enhancing the quality of NCD care in primary health centers. These interventions targeted both patients and physicians and were led by non-governmental actors. Two themes were noted within these publications. First, eHealth tools were commonly used as convenient methods for engaging with patients and clinicians to advance education, behavior change and adherence to guidelines. Second, the policy of integrating refugees into host-country health systems was uncommon and where implemented, described in sparse detail. This is evidenced by the limited number of policies identified that incorporated refugees into national health systems. In the scoping review results, integration existed on a spectrum from full inclusion in host-country health systems to calls for NGO actors to utilize their resources to strengthen local health systems by incorporating refugee health workers into service provision. Both themes are discussed further below.

##### Utilizing e-health tools to improve healthcare quality

In 2009, UNRWA embarked on a series of improvements to their health services, with specific attention to diabetes and hypertension. This reform was documented in more than six peer-reviewed publications. Three of these studies were included in the scoping review because they emphasized separate segments of the reform [22–24]. The most recent publication [22] described the implementation journey from 2009 to 2017, when the vast majority of UNRWA health centers completed the rollout. The first two segments of the intervention were the electronic medical record (EMR) and the development of family health teams. The EMR was web-based, utilized the International Classification of Disease codes (ICD 10), and had a built-in appointment system as well as several other clinical functions. The second component of this reform -- family health teams, included a restructuring of services to provide comprehensive primary healthcare and connect families to long term support from a team of providers. The reform also included an mHealth component that addressed specific issues for mothers and children [22]. The authors presented three indicators as evidence of the interventions progress -- a reduction in physician consultations, a reduction in antibiotic prescription rates and high physician satisfaction with the EMR.

This reform was further expanded upon in 2 publications by Khader et al. (2012), which highlighted the cohort monitoring of Palestinian refugees with diabetes and hypertension in one clinic in Jordan, the Nuzha Primary Health Care Clinic [23, 24]. The authors argued that cohort monitoring-- the frequent review of reports on treatment and outcomes for a particular group of patients--could inform quality improvement efforts over time. Several cohorts were monitored using the EMR system, in order to understand the basic demographics of the patient population. Monitoring also identified program performance on indicators such as the percentage of diabetic patients who had had their blood glucose measured and the percentage of diabetic patients who had received a foot exam. The authors discovered that the clinic performed poorly on several indicators, with just 42% of diabetic patients having had their blood glucose measured as well as little effort to conduct foot exams and no evidence of eye exams. The data showed that cohort monitoring had the potential to aid clinicians in identifying problems and root causes of poor quality care. However, no actions were taken to change the poor performance, though the authors suggested how the data could be utilized in the future.

An intervention in Lebanon also used electronic health tools to improve the quality of care provided in ten primary health centers, managed by the International Organization for Migration and the International Medical Corps. A longitudinal cohort study was conducted to improve care for Syrian refugees and Lebanese patients through the implementation of clinical guidelines and the adoption of an mHealth application [25]. The guidelines were adapted for the local context, and clinicians were trained on all components of the protocol. The mHealth application was implemented as both an EMR for clinicians and a personally-controlled health record (PCHR) for patients. Physicians utilized the EMR for documentation of patient care, and patients used the PCHR component to increase the mobility of their record and to access educational materials on medications and lifestyle behaviors. The full program was implemented in two phases over 20 months.

The authors measured several outcomes: clinical measurements (i.e., blood pressure), patient-provider interactions (measured by exit interviews) and medication prescription and use (measured by phone interviews and health records). Findings were mixed and highlighted that guidelines alone did not improve outcomes, whereas guidelines plus an mHealth application improved several outcomes. Changes in clinical measures were not significant, though the authors argued that the implementation period was short and that many clinicians rejected use of the application. All components of patient-provider interactions improved significantly (i.e., the provider took a medical history, the provider asked about medication complications) and there were notable increases in the reporting of medication prescription and use in the EMR [25].

##### Integrating refugees into host country health systems

In addition to the use of eHealth applications to improve NCD services, other actors attempted to integrate refugee health workers or refugee health services into host-country health systems. For example, Medical Teams International (MTI) was an early responder to the health needs of Syrian refugees in Lebanon. MTI provided mobile clinics and then shifted service delivery to a focus on expanding the role of community health workers [26]. Several factors motivated MTI’s change in approach. First, the Lebanese government required that NGOs invest in health systems strengthening rather than develop parallel health structures. Second, MTI conducted several studies that identified key gaps in service provision in their own programs. The organization responded by investing in refugee outreach volunteers (ROVs) who served as community health workers. ROVs monitored disease control for community members with diabetes and hypertension, led discussions on changes to diet and smoking habits, conducted cardiovascular disease risk assessments and referred high-risk refugees to primary health centers (PHCs). The authors reported descriptive statistics, including the number of blood pressure monitoring visits completed by ROVs and the number of refugees referred to PHCs for more advanced care. The intervention was not measured for effectiveness and did not present outcome indicators.

One study aimed to improve access to care for refugees with end-stage renal disease in Iran. The Iranian national health policy provided Afghan refugees access to dialysis and kidney transplantation at government facilities [27]. Specifically, Afghan refugees, with end-stage renal disease (ESRD) could receive kidney donations from others of the same nationality. In 2004, the authors conducted a simple descriptive analysis of the transplantation program in Tehran, through a review of Ministry of Health (MOH) records. They found that 241 refugees had ESRD, 179 were on dialysis and 62 had received a transplant. It should be noted that Afghan refugees could not be kidney donors to Iranian nationals for fear of exploitation or coercion. The integration of Afghan refugees into the Iranian health system was a novel approach to managing the health and wellness of displaced persons. While this integration was not measured for effectiveness, it was monitored over time to document availability of the service and any risk of ethical concerns.

#### Theme 3: preventing NCDs and NCD complications

Prevention efforts were highlighted in three of the identified studies. Collins et al. [28] illustrated a cardiovascular disease (CVD) risk assessment and prevention program that took place in two Médecins Sans Frontières (MSF) clinics in Jordan. The clinics introduced CVD risk assessments in tandem with cholesterol testing. This risk assessment tool was applied to Syrian refugees and Jordanian patients with hypertension, diabetes, chronic obstructive pulmonary disease, asthma and CVD and was intended to aid clinicians in identifying risk and prescribing relevant medications. The team conducted a mixed-methods study to understand the extent to which the tool was used and challenges faced during implementation. Quantitative findings revealed that very few patients had a CVD risk score assigned to them and that half of the high-risk patients were not prescribed the needed medication. Qualitative findings uncovered reasons for this failure to adhere to the CVD risk assessment guidelines, including confusion about how to use risk assessment charts and the desire to prioritize lifestyle changes over medications. Further, the risk assessment was only being conducted by physicians but several nurses seemed to have a better understanding of the tool. This intervention focused on improving clinician behavior and preventing CVD among patients who had other NCD diagnoses.

The remaining two prevention efforts aimed to change patient behavior. Rowther et al. [29] presented a diabetes risk assessment and motivational interviewing intervention in a Jordanian clinic managed by the Institute for Family Health (IFH). This program, the Computer-Assisted Diabetes Risk Assessment and Education program (CADRAE), targeted marginalized communities across a broad spectrum including refugees (Syrian, Palestinian and Iraqi) and Jordanians. The initiative had two components: a self-administered computerized survey that helped patients identify their diabetes risk, followed by a short, computerized, motivational interview that supported patients in considering lifestyle changes. The survey portion asked patients about family history, use of anti-hypertensives, physical activity, fruit and vegetable intake, body mass index and other items. The motivational interview was a mini-counseling session aimed at aiding patients in setting achievable goals around their behaviors. Both activities took place in the IFH clinic waiting room. Patients also received phone calls 1-month after the encounter. Program effectiveness was not evaluated.

Abu Kishk et al. [30] evaluated the final prevention-related intervention, a community-based campaign for Palestinian refugees who had diabetes and attended an UNRWA clinic. The six-month campaign, “Life is Sweeter with less Sugar” included education sessions focused on, among other things, diabetic symptoms, medications and dental care. The campaign also incorporated monthly cooking classes and bimonthly exercise sessions in various communal locations. Similar to most UNRWA interventions, this campaign took place in four out of five of UNRWA’s locations -- Jordan, the West Bank, Lebanon and Gaza. Syria was excluded as a result of the Syrian Civil War. The authors evaluated the effectiveness of the intervention by analyzing pre-and-post performance on several data elements, including demographics, body measurements, blood tests and blood pressure. Significant changes were observed in all areas.

## Discussion

This scoping review identified 11 publications that presented interventions and policies aimed at addressing NCDs among urban refugee populations in MENA region. Ten of the 11 publications focused on interventions, and only one described a national policy. The majority of the studies were conducted in Jordan, addressed the NCD needs of adults, and tackled five diseases - diabetes, hypertension, cancer, cardiovascular disease and end-stage renal disease. Palestinian and Syrian refugees were the most common population targeted for support. However, the majority of papers that focused on Palestinians pertained to a single regional reform effort undertaken by a UN agency, UNRWA. Other funding and implementing organizations involved in the selected studies were Médecins Sans Frontières, International Organization for Migration, International Medical Corps, the Transplantation Unit at Hashemi Nejad Kidney Hospital, Institute for Family Health, Noor Al Hussein Foundation, University of California-Irvine, American University of Beirut, Children’s Cancer Center of Lebanon Foundation, St. Jude Children’s Research Hospital, the American Lebanese Syrian Associated Charities and Medical Teams International.

It is important to note the paucity of published work that thoroughly described NCD interventions and policies related to urban refugees in crisis-affected contexts or evaluated the efficiency and effectiveness of such approaches. In light of the growing burden of noncommunicable diseases in middle-income settings, coupled with the increasing frequency of humanitarian crises in middle-income countries, the field requires greater investment in research on effective ways of addressing NCDs. Much of the peer-reviewed literature articulated the challenges faced -- high prevalence, high percentage of deaths due to NCDs, general barriers to care and other upstream concerns – but presented very few tested solutions.

This review produced five key findings that have implications for research and practical efforts to address NCDs among urban refugees in the Middle East and North Africa. First, very few studies focused on the prevention of noncommunicable diseases among displaced populations. This is unfortunate since displacement is increasingly a long-standing situation, and prevention is a pillar of efforts to improve long-term health. Primary prevention efforts are less expensive than treatment and can shift the focus from expensive hospital services to less expensive health centers and community-based programs [31]. The studies identified through this review focused solely on patients who had already been diagnosed with a disease and were being treated at a primary healthcare clinic. Only one study implemented a program outside of a clinic setting and incorporated community-based concepts. However, that study team focused only on patients who had been diagnosed in their primary healthcare clinics, and were thus attempting to prevent secondary complications.

Opportunities exist to engage in the primary prevention of noncommunicable diseases among adults, adolescents and children who have yet to be affected by any diagnosis. Greater attention and funding should be directed towards primary prevention in order to reduce morbidity, mortality and healthcare costs [7]. Moreover, many NCDs are preventable through changes in individual behavior, reductions in social and economic inequalities and regulation of commercial determinants of disease. Two of the three prevention studies focused on the former, changing patient behavior, while the third prioritized modifying physician behavior. No studies explored more complex root causes of NCDs, or implemented multi-sectoral approaches to address these issues. Prevention efforts could include education, smoking cessation, cooking courses, the modification of public spaces to improve accessibility and legislation that limits the marketing of unhealthy foods. In addition, several interventions highlighted in this review may be adapted to address prevention concerns, including the deployment of refugee outreach volunteers and the utilization of mobile technologies for education and coaching.

Second, a promising area of intervention was the use of health information technologies (HIT), including electronic medical records (EMRs), NCD databases for clinicians, and mHealth applications for patients. EMRs were used to manage daily clinical encounters as well as monitor patients over time to identify trends in care and areas for improvement in service delivery. An NCD database was utilized to review physician practice behaviors and identify the need for additional intervention with physicians and nurses. An mHealth application, administered on a portable touch-screen computer, helped patients understand their risk of developing diabetes and encouraged them to modify their lifestyle to avoid diagnosis. One intervention combined an EMR for clinicians with a personally controlled health record (PCHR) for patients, providing both parties access to the medical record, the ability to move the record easily to another facility, and to view educational materials. The use of HITs in resource-poor settings has increased dramatically in recent years and has been used to manage a variety of health concerns and health systems challenges [7, 32]. HIT may be well suited for prevention, treatment and rehabilitation efforts among populations on the move. Health and humanitarian actors should continue to research and expand on these experiences with HITs in order to identify effective interventions and scale them up in relevant contexts.

Third, the vast majority of publications were solely focused on tackling NCDs through primary care efforts and did not address specialized needs or services. While primary care is a key component of NCD services, access to specialists and more advanced care is important for the prevention of mortality and morbidity. For example, people with diabetes are at risk of diabetic retinopathy and neuropathy, and specialists help to treat and avoid these secondary complications. Primary care interventions were the most common because the implementers identified in this review were NGOs and UN actors. As a result of funding limitations and a lack of experience with supporting NCDs in crisis settings, NGO and UN actors often only provide primary care. However, a prioritization of primary care without the support of specialists (i.e., endocrinologists, nephrologists, oncologists, pulmonologists) and secondary care settings reduces access to comprehensive and coordinated NCD services and negatively impacts health outcomes. Access to advanced secondary and tertiary NCD services are an urgent concern among refugee populations in urban/non-camp settings and likely can only be improved through efforts to integrate refugee populations into national health systems [33].

Fourth, only one publication addressed a national health policy and did so with very little detail. Policy frameworks serve as guiding documents for how to address large scale health needs, include all populations, identify financial and other resources, cultivate partnerships and monitor and evaluate policy implementation. The dearth of documentation about policy frameworks that address NCD needs among refugees in urban settings is problematic. Policy development and implementation are complex and require the involvement of many stakeholders. Frequently key populations are left out of policies, or the steps taken to implement a policy lack fidelity to what the policy had intended to accomplish. Poorly crafted policies can discriminate against marginalized communities or have other unintended consequences. As a result, continuous, unbiased monitoring and evaluation of national policies is key to ensuring inclusiveness and effectiveness. Greater efforts are needed to document and evaluate current policy frameworks and their success in addressing NCDs in displaced populations.

Moreover, the one policy identified in this scoping review responded to the needs of Afghan refugees by making them eligible for Iran’s national program for dialysis and kidney transplantation. This policy focused on the integration of refugees into host country health systems. UNHCR and other actors tout integration as the best approach to meeting the needs of displaced persons in urban settings, particularly because of the protracted nature of crises. However, the execution of this philosophy has been limited, and where it has occurred, description and measurement have been minimal [34, 35]. Rather than integrating refugees into host country health systems, many actors provide health services for refugees through parallel programs and structures. The problems that arise as a result of parallel service delivery have been widely discussed, including limited sustainability and wastage of resources [14, 34, 36]. Parallel structures also risk doing harm by creating short term programs that are frequently interrupted, providing services that are not aligned with cultural expectations, and focusing heavily on treatment over prevention of illness [37]. Documentation and evaluation of policy frameworks could aid stakeholders in exploring integration opportunities and determining feasibility and effectiveness of such approaches.

Last, refugee perspectives on their own health and their access to NCD services were notably absent from the literature. While interventions and policies were presented in varying depth and quality, researchers failed to document how refugees interacted with health services in urban settings and whether these services met their needs. Capturing and analyzing the experiences of affected persons is key to increasing health access, enhancing the quality of services and improving health outcomes. Dozens of authors have argued that health professionals and health systems benefit from listening to patients and communities [38, 39]. This ‘listening’ allows the system to respond to needs and engage patients in the co-creation and design of health services and other approaches to wellness [38, 40]. Incorporating patient and community perspectives into the design and evaluation of programs results in a variety of benefits including empowerment of vulnerable communities, strengthening of health systems and greater patient self-care and self-management [39]. This gap in the literature suggests that clinicians and health systems may have a limited understanding of patient and community assets and needs, and may be making incorrect assumptions about what is most useful for the populations they serve.

### Limitations

These findings should be viewed in light of several limitations. This scoping review included all possible study methodologies and did not attempt to critique the quality of the selected studies. However, this is aligned with the goal of scoping reviews and made it possible to review a wide range of interventions and policies from across the region. Also, the review is limited to select years (2000–2018) and the English language, which may have resulted in missed opportunities to identify novel approaches to NCDs. However, it is recognized that the discourse on tackling NCDs among urban refugees increased in response to the fleeing of Iraqi refugees in 2003 and Afghan refugees in 2001. Thus, the majority of the reviewed work on this topic occurred in this time period.

There is a risk that the works captured by this review do not include all relevant interventions and policies, particularly because practitioners may not have published these items as peer-reviewed literature and grey literature was not reviewed. Further, in this scoping review, each intervention and policy was described at differing levels of depth due to differences in depth of description and analysis in the original publication. A future review may benefit from a more thorough consultative process, wherein reviewers contact and interview implementers to gain more in-depth understanding of intervention characteristics and additional findings that may have arisen post-publication. Even with these limitations, the findings of this study have significant implications for practitioners, policymakers and donors and can be utilized to explore additional research questions, identify possible interventions worth piloting and collaborate with actors that have valuable experience in the subject area.

## Conclusion

The aim of this review was to compile research about the interventions and policies aimed at addressing the needs of urban based refugees diagnosed with noncommunicable diseases across the MENA region. The review concluded that, (1) very few interventions were aimed at preventing NCDs among the forcibly displaced, (2) that eHealth and mHealth were readily used across different crisis settings and (3) that most NCD efforts solely focused on primary care, while secondary and tertiary NCD care were absent from the refugee literature. Additionally, the review identified (4) limited writing on health policies and (5) an absence of work that inquired about refugee perspectives and experiences with NCD services in host countries.

Much additional work is needed to provide comprehensive, equitable, quality health supports for urban refugee populations. In regard to NCDs, opportunities exist to prioritize prevention efforts, scale up eHealth and mHealth interventions, expand access to secondary and tertiary services, analyze national health policies and elevate the voices of refugees in health services research. While there are several interventions and policies that appear promising, other efforts will require more rigorous study designs to determine effectiveness in diverse settings. This scoping review is a first step in documenting current interventions and policies and recognizing strengths and gaps across these approaches. Practitioners, policymakers and donors can utilize this content to more strategically plan local, national and global responses to NCD needs among refugees residing in urban settings.

## Supplementary information

**Additional file 1.** Search syntax.

## Data Availability

Not applicable.

## Abbreviations

CADRAE: Computer-assisted diabetes risk assessment and education
COPD: Chronic obstructive pulmonary disease
CVD: Cardiovascular disease
DM: Diabetes mellitus
ECC: Exceptional care committee
EMR: Electronic medical record
ESRD: End-stage renal disease
HIT: Health information technology
ICD: International Classification of Diseases and Related Health Problems
IFH: Institute for Family Health
IOM: International Organization for Migration
MOH: Ministry of Health
MSF: Médecins Sans Frontières
NCD: Noncommunicable disease
NGO: Non-governmental organization
PCHR: Patient-controlled health record
PEN: Package of Essential noncommunicable disease interventions
PHC: Primary health center
ROV: Refugee outreach volunteer
UN: United Nations
UNHCR: The UN Refugee Agency
UNRWA: United Nations Relief and Works Agency for Palestine Refugees in the Near East
WHO: World Health Organization
