# Supplementary material for: Addressing noncommunicable diseases among urban refugees in the Middle East and North Africa - a scoping review
Source: Confl Health. 2020 Feb 18;14:9. doi: 10.1186/s13031-020-0255-4 (PMC7029555; doi:10.1186/s13031-020-0255-4)
Supplement: Supplementary file 1 — Additional file 1. Search syntax. [file 13031_2020_255_MOESM1_ESM.docx]

**Appendix 1. Search syntax**

(((("Refugees"[tiab]) OR "Refugees"[Mesh])) AND ((((((((("Iran"[tiab]) OR "Iran"[Mesh]) OR "Turkey"[tiab]) OR "Turkey"[Mesh]) OR "Jordan"[tiab]) OR "Jordan"[Mesh]) OR "Lebanon"[tiab]) OR "Lebanon"[Mesh]) OR (("Middle East"[tiab]) OR "Middle East"[Mesh]))) AND ((((((((((((("chronic respiratory disease"[tiab]) OR "Chronic Disease"[tiab]) OR "Chronic Disease"[Mesh]) OR "Noncommunicable Diseases"[tiab]) OR "Noncommunicable Diseases"[Mesh]) OR "Neoplasms"[tiab]) OR "Neoplasms"[Mesh]) OR "Hypertension"[tiab]) OR "Hypertension"[Mesh]) OR "Cardiovascular Diseases"[tiab]) OR "Cardiovascular Diseases"[Mesh]) OR "Diabetes Mellitus"[tiab]) OR "Diabetes Mellitus"[Mesh])
